# *β*-Cell Expansion for Therapeutic Compensation of Insulin Resistance in Type 2 Diabetes

**DOI:** 10.1080/15438600303731

**Published:** 2003

**Authors:** Shimon Efrat

**Affiliations:** 1 Department of Human Genetics and Molecular Medicine Sackler School of Medicine Tel Aviv University Ramat Aviv Tel Aviv 69978 Israel

## Abstract

Insulin resistance is the primary cause of type 2 diabetes. However, if compensated by increased insulin production, insulin resistance by itself does not lead to overt disease. Type 2 diabetes develops when this compensation is insufficient, due to defects in *β*-cell function and in regulation of the *β*-cell mass. *β*-Cell transplantation, as well as approaches that replenish or preserve the endogenous *β*-cell mass, may facilitate the treatment of type 2 diabetes in patients requiring exogenous insulin.


*Experimental Diab. Res.*, 4:1–5, 2003
Copyright © 2003 Taylor & Francis
1543-8600/03 $12.00 + .00
DOI: 10.1080/15438600390214635

Taylor & Francis healthsciences

### $\beta$-Cell Expansion for Therapeutic Compensation of Insulin Resistance in Type 2 Diabetes

**Shimon Efrat**

*Department of Human Genetics and Molecular Medicine, Sackler School of Medicine, Tel Aviv University, Tel Aviv, Israel*

**Insulin resistance is the primary cause of type 2 diabetes. However, if compensated by increased insulin production, insulin resistance by itself does not lead to overt disease. Type 2 diabetes develops when this compensation is insufficient, due to defects in $\beta$-cell function and in regulation of the $\beta$-cell mass. $\beta$-Cell transplantation, as well as approaches that replenish or preserve the endogenous $\beta$-cell mass, may facilitate the treatment of type 2 diabetes in patients requiring exogenous insulin.**

**Keywords** Apoptosis; $\beta$-Cell Transplantation; Insulin Production and Secretion; Reversible Immortalization; Stem Cell Differentiation

Type 2 diabetes is a genetic disease aggravated by obesity [1] and a sedentary lifestyle. Insulin resistance is thought to be the primary metabolic abnormality in type 2 diabetes. However, if compensated by increased insulin production, insulin resistance itself does not lead to diabetes. In obese nondiabetic people, the pancreatic islet $\beta$ cells respond to insulin resistance by increased insulin biosynthesis and secretion [2]. In addition, insulin output is regulated by expansion of the $\beta$-cell mass, which has been observed in both obese nondiabetic people [3] and rodent models of obesity. In patients with type 2 diabetes, the compensatory effect of $\beta$ cells is insufficient. This may result both from functional impairments in glucose-induced insulin secretion from $\beta$ cells [4], as well as abnormalities in maintenance and expansion of the $\beta$-cell mass. The $\beta$-cell mass is the net result of a number of dynamic processes, including $\beta$-cell replication, islet neogenesis from pancreatic ducts, and $\beta$-cell death [5]. A recent analysis of pancreatic tissue from autopsies of patients with type 2 diabetes documented a significant decrease in $\beta$-cell mass [3]. The findings suggested that islet neogenesis was normal in type 2 diabetes, while the rate of $\beta$-cell apoptosis was significantly increased, compared with nondiabetic controls [3]. Thus, instead of showing an increase in $\beta$-cell mass, aimed at compensation for insulin resistance, patients with type 2 diabetes manifest a reduction in $\beta$-cell mass, compared with normal subjects.

Current therapy of type 2 diabetes includes changes in diet and exercise, as well as the use of drugs that increase insulin secretion, reduce hepatic glucose output, and increase target cell sensitivity to insulin. Eventually, administration of exogenous insulin is required to normalize blood glucose in a significant number of patients. Expansion of the $\beta$-cell mass by stimulation of $\beta$-cell replication or neogenesis, by protection of $\beta$ cells from apoptosis, as well as by $\beta$-cell transplantation, may facilitate the treatment of type 2 diabetes.

### REPLENISHMENT AND PRESERVATION OF THE ENDOGENOUS $\beta$-CELL MASS

The emerging understanding of embryonic $\beta$-cell development [6], and of $\beta$-cell regeneration in the adult pancreas from pancreatic ducts [7], may hold the promise of developing approaches to stimulate endogenous $\beta$-cell replenishment by neogenesis or replication. These approaches are likely to involve the local delivery of growth factors or other extracellular

Received and accepted 20 January 2003.
Address correspondence to Shimon Efrat, Department of Human Genetics and Molecular Medicine, Sackler School of Medicine, Tel Aviv University, Ramat Aviv, Tel Aviv, 69978 Israel. E-mail: sefrat@post.tau.ac.il mitogenic agents, as well as the transfer of genes into pancreatic duct or islet cells that can modulate their replication and differentiation, or increase their resistance to apoptosis. Although these prospects await the development of safe and efficient methods for local delivery of proteins and genes, it is possible that systemic treatment with growth factors may lead to a significant expansion of the $\beta$-cell mass without deleterious effects to other tissues. One example for such a factor is exendin-4, a stable analog of glucagon-like peptide 1 (GLP1), which has been shown to stimulate both $\beta$-cell neogenesis and replication in a rat model of type 2 diabetes involving partial pancreatectomy [8]. In a recent work using Goto-Kakizaki rats, a nonobese model of type 2 diabetes, injections of GLP1 or exendin-4 increased the $\beta$-cell mass, resulting in long-term improvements in glycemia [9]. GLP-1 is a particularly attractive candidate because of its additional stimulatory effects on glucose-induced insulin secretion from $\beta$ cells [10]. Numerous other hormones and growth factors are known to stimulate duct cell differentiation into islets and $\beta$-cell replication (see [11] for a recent review); however, most of them are unlikely to be suitable for systemic treatment due to pleotropic effects in other tissues.

### $\beta$-CELL TRANSPLANTATION

The alternative to stimulating the replenishment of the endogenous $\beta$-cell mass is increasing it from an exogenous source, by transplantation of alloegneic $\beta$ cells. The challenges facing $\beta$-cell transplantation in type 2 diabetes are less daunting, compared with type 1 diabetes. Although $\beta$ cells transplanted into patients with type 2 diabetes may be exposed to an environment that could impair their function or viability, these factors are much less potent and slower to act, compared with recurring autoimmunity facing $\beta$-cell transplantation in type 1 diabetes. In addition, the effect of these factors may decrease with improvement in glycemia. Similarly, although regulated insulin secretion is a critical requirement from cells considered for transplantation into patients with type 1 diabetes, this is not a consideration in type 2 diabetes, where large amounts of insulin are needed to overcome insulin resistance.

Allogeneic $\beta$-cell transplantation on a large scale cannot rely on scarce human islet donors to provide cells for the large population of patients with type 2 diabetes who may benefit from transplantation. It will require the development of new and abundant sources of $\beta$ cells, based on efficient ways for expansion of $\beta$ cells in tissue culture. This expansion of the cell mass should be achieved without a loss of the $\beta$-cell phenotype, as characterized by the amounts of insulin produced and stored in the cells, and its regulated secretion. Two different approaches have been taken in recent years toward $\beta$-cell expansion in tissue culture: (1) stimulation of replication of mature $\beta$ cells and (2) induction of differentiation of progenitor cells from various sources into cells with $\beta$-cell properties.

### $\beta$-Cell Expansion by Reversible Immortalization

Because mature $\beta$ cells do not divide much in vitro, oncogene expression has been used to force their replication. Studies involving expression of oncogenes in $\beta$ cells of transgenic mice resulted in the development of $\beta$-cell lines, which proved valuable for investigating $\beta$-cell function and gene expression [12]. However, induction of forced replication of postmitotic $\beta$ cells ultimately lead to impaired insulin production and secretion in these cell lines. The phenotypic instability, as well as the uncontrolled growth, made this approach incompatible with a therapeutic application.

These shortcomings were overcome by the development of conditional gene expression systems, which allowed reversible regulation of oncogene expression. Expression of SV40 large T antigen (Tag) oncoprotein in $\beta$ cells in transgenic mice under control of the tet-off and tet-on systems resulted in the generation of differentiated $\beta$-cell lines with a stable phenotype [13–15]. Such cells could be expanded in tissue culture by oncogene expression, and induced to undergo growth arrest in its absence, using tetracycline (tet) ligands. Following growth arrest, the cells increased their insulin storage severalfold, up to the levels typical of normal mouse islets, and maintained normal regulation of insulin secretion in response to glucose in the physiological concentration range [14, 15]. When transplanted into streptozotocin-induced diabetic syngeneic mice, these cells restored euglycemia and maintained it for long periods of time [13, 15]. Using the tet-on approach had the advantage of avoiding the need to treat transplanted animals with tet ligands following transplantation to prevent oncogene expression and undesired cell expansion.

Unfortunately, application of reversible immortalization strategies to human $\beta$ cells proved to be difficult. Immortalized human $\beta$ cells tend to lose differentiation much faster than mouse $\beta$ cells. Strategies for restoring the function of dedifferentiated human $\beta$ cells, for example by expression of insulin gene transcription factors, resulted in a partial recovery of insulin production [16]; however, the phenotypic stability of such cells in tissue culture or following transplantation has not been determined. Despite effective methods for oncogene silencing or removal, concerns have been expressed about the oncogenic potential of such cells. Additional safety elements, such as using physical barriers in the form of semipermeable membrane devices, or cell engineering with suicide genes to allow cell elimination in case of device failure, will be needed to address these concerns.

### Development of Insulin-Producing Cells From Embryonic or Tissue Stem Cells

The alternative to expansion of mature β cells is differentiation of stem or progenitor cells into surrogate β cells. Stem cells possess a natural replication capacity in tissue culture. When allowed to spontaneously differentiate, both murine and human embryonic stem (ES) cells give rise to a low percentage of insulin-producing cells [17, 18]. Initial efforts were directed toward isolation of these relatively rare cells using selection procedures. Subsequently, protocols were developed to increase the fraction of ES cells that develop into insulin-producing cells. McKay and coworkers selected nestin-positive neuroendocrine precursor cells, which developed from mouse ES cells, and utilized combinations of soluble factors to promote their differentiation in tissue culture into islet cell types [19]. Hori and colleagues treated mouse ES cells with inhibitors of phosphoinositide 3-kinase, thereby generating cells that produced significant insulin levels and released it in response to glucose [20]. Although insulin-producing cells developed from ES cells were able to normalize glycemia in mice, the ability of such cells derived from human ES cells to replace the function of differentiated β cells in humans remains unknown.

In addition to stem cells derived from early-stage embryos, evidence suggests that many fetal and adult tissues contain immature cells, which are responsible for tissue renewal. Such cells maintain a replicative capacity and an ability to differentiate into a number of cell types. The most obvious place to look for cells that can potentially differentiate into insulin-producing cells is the pancreas. Duct cells can form islet-like structures in culture [21, 22]; however, they are difficult to expand. The isolation and characterization of the pancreatic islet stem cells remains a goal for future efforts.

In recent years, the cell commitment dogma has been challenged by reports that progenitor cells from adult organs can give rise to unrelated cell types, both in vivo and in culture. It is unclear at present whether these findings represent bona fide “transdifferentiation,” or the persistence of residual embryonic pluripotent cells in adult tissues. The most striking example is bone marrow cells from both mice and humans, which were shown to give rise to a diverse range of cell types from other tissues [23]. Although the natural plasticity of tissue stem cells is still being debated, there is evidence that progenitor cells committed to develop into certain tissues can be at least partly reprogrammed with dominant genes that activate a cascade of developmental events. For example, expression of a homeobox transcription factor, Pdx1, which plays key roles in pancreas development and gene expression in mature β cells, in mouse liver cells in vivo [24], and in rat enterocytes in vitro [25], was shown to activate β-cell genes, including insulin. Liver progenitor cells represent an attractive source, because liver and pancreas share embryological origin from the primitive foregut. In addition, mature hepatocytes and β cells manifest similarities in gene expression profiles, such as transcription factors, the glucose transporter GLUT2, and the glucose-phosphorylating enzyme glucokinase. Progenitor cells cultured from mouse fetal liver were shown to be pluripotent and differentiate in vivo into a number of hepatic, pancreatic, and intestinal cell types [26]. Furthermore, adult rat hepatic stem cells, termed oval cells, were shown to differentiate into pancreatic endocrine cells in vitro [27]. New insights into the cascade of transcription factors that act during endocrine pancreas development [6] will allow the employment of combinations of such factors in an effort to direct the differentiation of liver and other tissue stem cells into β-like cells.

### CONCLUDING REMARKS

Increasing the β-cell mass is likely to improve glycemia and replace insulin administration in patients with type 2 diabetes. Although progress has been made in our understanding of genes involved in β-cell development and factors that regulate β-cell differentiation, replication, and survival, approaches for replenishment or preservation of endogenous β cells in humans are not available at present. In contrast, transplanted β cells, which can be genetically manipulated in tissue culture to improve their function and survival, may represent a more realistic approach. Recent progress in β-cell expansion in tissue culture and the formation of insulin-producing cells from stem and progenitor cells holds the promise of developing abundant cell sources for transplantation. Another important consideration, beyond technical feasibility, is the functional performance of endogenous versus transplanted cells. It is not known whether the functional abnormalities observed in β cells in type 2 diabetes, as well as the increased rate of apoptosis, are caused by genetic defects or by epigenetic factors, such as the chronic hyperglycemia and deposits of islet amyloid polypeptide (IAPP), which characterize the β-cell milieu in type 2 diabetes. Although the effects of these factors may take a long time to manifest themselves, in both cases β-cell transplantation may be more advantageous, compared with replenishment or preservation of endogenous β cells. If epigenetic conditions are the main cause of β-cell failure in type 2 diabetes, autologous and allogeneic new β cells will be equally susceptible to a gradual deterioration in their function and viability. However, genetic engineering of the transplanted cells with genes involved in insulin production and secretion, as well as antiapoptotic genes, may prolong their normal function and survival following transplantation. Such genetic manipulations are much harder to perform in cells formed endogenously, due to the difficulty of targeting viral vectors to the pancreas. In the event that intrinsic $\beta$-cell abnormalities originating from genetic defects are the primary cause of impaired $\beta$-cell function and increased apoptosis in type 2 diabetes, new $\beta$ cells formed endogenously may deteriorate faster, compared with transplanted allogeneic cells. Thus, when cell function alone is considered, it may be preferable to increase the $\beta$-cell mass using an exogenous, rather than an endogenous, source of $\beta$ cells. On the other hand, the risks of immunosuppression, as well as the challenges of achieving appropriate vascularization and regulation of $\beta$ cells placed outside the pancreas, are considerations against cell transplantation. Moreover, before proceeding with any therapy for type 2 diabetes that is based on cell manipulation, its risks will have to be weighed against the improvement in glycemia, in comparison with that achieved with simple insulin administration.

### REFERENCES

[1] Ludvik, B., Nolan, J. J., Baloga, J., Sacks, D., and Olefsky, J. (1995) Effect of obesity on insulin resistance in normal subjects and patients with NIDDM. *Diabetes,* **44,** 1121–1125.

[2] Polonsky, K. S. (2000) Dynamics of insulin secretion in obesity and diabetes. *Int. J. Obes. Relat. Metab. Disord.,* **24**(Suppl. 2), S29–S31.

[3] Butler, A. E., Janson, J., Bonner-Weir, S., Ritzel, R., Rizza, R. A., and Butler, P. C. (2003) Beta-cell deficit and increased beta-cell apoptosis in humans with type 2 diabetes. *Diabetes,* **52,** 102–110.

[4] Leahy, J. L. (1990) Natural history of beta-cell dysfunction in NIDDM. *Diabetes Care,* **13,** 992–1010.

[5] Finegood, D. T., Scaglia, L., and Bonner-Weir, S. (1995) Dynamics of beta-cell mass in the growing rat pancreas. Estimation with a simple mathematical model. *Diabetes,* **44,** 249–256.

[6] Schwitzgebel, V. M. (2001) Programming of the pancreas. *Mol. Cell. Endocrinol.,* **185,** 99–108.

[7] Wang, R. N., Kloppel, G., and Bouwens, L. (1995) Duct-to-islet-cell differentiation and islet growth in the pancreas of duct-ligated adult rats. *Diabetologia,* **38,** 1405–1411.

[8] Xu, G., Stoffers, D. A., Habener, J. F., and Bonner-Weir, S. (1999) Exendin-4 stimulates both beta-cell replication and neogenesis, resulting in increased beta-cell mass and improved glucose tolerance in diabetic rats. *Diabetes,* **48,** 2270–2276.

[9] Tourrel, C., Bailbe, D., Lacorne, M., Meile, M.J., Kergoat, M., and Portha, B. (2002) Persistent improvement of type 2 diabetes in the Goto-Kakizaki rat model by expansion of the beta-cell mass during the prediabetic period with glucagon-like peptide-1 or exendin-4. *Diabetes,* **51,** 1443–1452.

[10] Kieffer, T. J., and Habener, J. F. (1999) The glucagon-like peptides. *Endocr. Rev.,* **20,** 876–913.

[11] Nielsen, J. H., Galsgaard, E. D., Moldrup, A., Friedrichsen, B. N., Billestrup, N., Hansen, J. A., Lee, Y. C., and Carlsson, C. (2001) Regulation of beta-cell mass by hormones and growth factors. *Diabetes,* **50**(Suppl. 1), S25–S29.

[12] Efrat, S., and Fleischer, N. (2000) Engineering the pancreatic $\beta$-cell. In: *Diabetes Mellitus: A Fundamental and Clinical Text,* 2nd ed. Edited by LeRoith, D., Taylor, S., and Olefsky, J. M., pp. 535–541. Philadelphia, Lippincott, Williams & Wilkins.

[13] Efrat, S., Fusco-DeMan, D., Lemberg, H., Emran, O. A., and Wang, S. (1995) Conditional transformation of a pancreatic $\beta$-cell line derived from transgenic mice expressing a tetracycline-regulated oncogene. *Proc. Natl. Acad. Sci. U. S. A.,* **92,** 3576–3580.

[14] Fleischer, N., Chen, C., Surana, M., Leiser, M., Rossetti, L., Pralong, W., and Efrat, S. (1998). Functional analysis of a conditionally-transformed pancreatic $\beta$-cell line. *Diabetes,* **47,** 1419–1425.

[15] Milo-Landesman, D., Surana, M., Berkovich, I., Compagni, A., Christofori, G., Fleischer, N., and Efrat, S. (2001) Correction of hyperglycemia in diabetic mice transplanted with reversibly-immortalized pancreatic $\beta$ cells controlled by the tet-on regulatory system. *Cell Transplant.,* **10,** 645–650.

[16] de la Tour, D., Halvorsen, T., Demeterco, C., Tyrberg, B., Itkin-Ansari, P., Loy, M., Yoo, S. J., Hao, E., Bossie, S., and Levine, F. (2001) Beta-cell differentiation from a human pancreatic cell line in vitro and in vivo. *Mol. Endocrinol.,* **15,** 476–483.

[17] Soria, B., Roche, E., Berna, G., Leon-Quinto, T., Reig, J. A., and Martin, F. (2000) Insulin-secreting cells derived from embryonic stem cells normalize glycemia in streptozotocin-induced diabetic mice. *Diabetes,* **49,** 157–162.

[18] Assady, S., Maor, G., Amit, M., Itskovitz-Eldor, J., Skorecki, K. L., and Tzukerman, M. (2001) Insulin production by human embryonic stem cells. *Diabetes,* **50,** 1691–1697.

[19] Lumelsky, N., Blondel, O., Laeng, P., Velasco, I., Ravin, R., and McKay, R. (2001) Differentiation of embryonic stem cells to insulin-secreting structures similar to pancreatic islets. *Science,* **292,** 1389–1394.

[20] Hori, Y., Rulifson, I. C., Tsai, B. C., Heit, J. J., Cahoy, J. D., and Kim, S. K. (2002) Growth inhibitors promote differentiation of insulin-producing tissue from embryonic stem cells. *Proc. Natl. Acad. Sci. U. S. A.,* **99,** 16105–16110.

[21] Ramiya, V. K., Maraist, M., Arfors, K. E., Schatz, D. A., Peck, A. B., and Cornelius, J. G. (2000) Reversal of insulin-dependent diabetes using islets generated in vitro from pancreatic stem cells. *Nat. Med.,* **6,** 278–282.

[22] Bonner-Weir, S., Taneja, M., Weir, G. C., Tatarkiewic, K., Song, K. H., Sharma, A., and O'Neil, J. J. (2000) In vitro cultivation of human islets from expanded ductal tissue. *Proc. Natl. Acad. Sci. U. S. A.,* **97,** 7999–8004.

[23] Jiang, Y., Jahagirdar, B. N., Reinhardt, R. L., Schwartz, R. E., Keene, C. D., Ortiz-Gonzalez, X. R., Reyes, M., Lenvik, T., Lund, T., Blackstad, M., Du, J., Aldrich, S., Lisberg, A., Low, W. C., Largaespada, D. A., and Verfaillie, C. M. (2002) Pluripotency of mesenchymal stem cells derived from adult marrow. *Nature,* **418,** 41–49.

[24] Ferber, S., Halkin, A., Cohen, H., Ber, I., Einav, Y., Goldberg, I., Barshack, I., Seijffers, R., Kopolovic, J., Kaiser, N., and Karasik, A. (2000) Pancreatic and duodenal homeobox gene 1

induces expression of insulin genes in liver and ameliorates streptozotocin-induced hyperglycemia. *Nat. Med.,* **6,** 568–572.

[25] Kojima, H., Nakamura, T., Fujita, Y., Kishi, A., Fujimiya, M., Yamada, S., Kudo, M., Nishio, Y., Maegawa, H., Haneda, M., Yasuda, H., Kojima, I., Seno, M., Wong, N. C., Kikkawa, R., and Kashiwagi, A. (2002) Combined expression of pancreatic duodenal homeobox 1 and islet factor 1 induces immature enterocytes to produce insulin. *Diabetes,* **51,** 1398–1408.

[26] Suzuki, A., Zheng, Yw., Kaneko, S., Onodera, M., Fukao, K., Nakauchi, H., and Taniguchi, H. (2002) Clonal identification and characterization of self-renewing pluripotent stem cells in the developing liver. *J. Cell Biol.,* **156,** 173–184.

[27] Yang, L., Li, S., Hatch, H., Ahrens, K., Cornelius, J. G., Petersen, B. E., and Peck, A. B. (2002) In vitro trans-differentiation of adult hepatic stem cells into pancreatic endocrine hormone-producing cells. *Proc. Natl. Acad. Sci. U. S. A.,* **99,** 8078–8083.

